# HACE1 blocks HIF1α accumulation under hypoxia in a RAC1 dependent manner

**DOI:** 10.1038/s41388-021-01680-1

**Published:** 2021-02-18

**Authors:** Busra Turgu, Fan Zhang, Amal El-Naggar, Gian Luca Negri, Melanie Kogler, Luigi Tortola, Fraser Johnson, Tony Ng, Amy Li, Donald Yapp, William Lockwood, Daniel Martinez, John M. Maris, Mads Daugaard, Josef M. Penninger, Christopher S. Hughes, Poul H. Sorensen

**Affiliations:** 1 Department of Molecular Oncology, British Columbia Cancer Research Centre, Vancouver, Canada; 2 Interdisciplinary Oncology Graduate Program, Faculty of Medicine, University of British Columbia, Vancouver, Canada; 3 Department of Urological Sciences, The Vancouver Prostate Centre, University of British Columbia, Vancouver, Canada; 4 Department of Pathology and Laboratory Medicine, University of British Columbia, Vancouver, BC Canada; 5 Department of Pathology, Faculty of Medicine, Menoufia University, Shibin El Kom, Egypt; 6 Canada’s Michael Smith Genome Sciences Centre, BC Cancer, Vancouver, BC Canada; 7 Institute of Molecular Biotechnology of the Austrian Academy of Sciences, Vienna, Austria; 8 Department of Biology, Institute of Molecular Health Sciences, ETH Zurich, Switzerland; 9 Department of Experimental Therapeutics, British Columbia Cancer Research Centre, Vancouver, BC Canada; 10 Department of Integrative Oncology, British Columbia Cancer Research Centre, Vancouver, Canada; 11 Department of Pathology, Children’s Hospital of Philadelphia, Philadelphia, PA USA; 12 Division of Oncology and Center for Childhood Cancer Research, Children’s Hospital of Philadelphia, Philadelphia, USA; 13 Department of Medical Genetics, Life Science Institute, University of British Columbia, Vancouver, BC Canada

**Keywords:** Metastasis, Cell signalling

## Abstract

Uncovering the mechanisms that underpin how tumor cells adapt to microenvironmental stress is essential to better understand cancer progression. The *HACE1* (HECT domain and ankyrin repeat-containing E3 ubiquitin-protein ligase) gene is a tumor suppressor that inhibits the growth, invasive capacity, and metastasis of cancer cells. However, the direct regulatory pathways whereby *HACE1* confers this tumor-suppressive effect remain to be fully elucidated. In this report, we establish a link between *HACE1* and the major stress factor, hypoxia-inducible factor 1 alpha (HIF1α). We find that HACE1 blocks the accumulation of HIF1α during cellular hypoxia through decreased protein stability. This property is dependent on HACE1 E3 ligase activity and loss of Ras-related C3 botulinum toxin substrate 1 (RAC1), an established target of HACE1 mediated ubiquitinylation and degradation. In vivo, genetic deletion of *Rac1* reversed the increased HIF1α expression observed in *Hace1*^–/–^ mice in murine KRas^G12D^-driven lung tumors. An inverse relationship was observed between HACE1 and HIF1α levels in tumors compared to patient-matched normal kidney tissues, highlighting the potential pathophysiological significance of our findings. Together, our data uncover a previously unrecognized function for the HACE1 tumor suppressor in blocking HIF1α accumulation under hypoxia in a RAC1-dependent manner.

## Introduction

The E3 ubiquitin-protein ligase *HACE1* gene was first identified as a tumor suppressor gene in sporadic Wilms’ tumor (WT), based on its location at a WT translocation breakpoint and loss of expression compared to the normal kidney [1, 2]. Full-length HACE1 protein consists of six N-terminal ankyrin repeats responsible for protein–protein interactions and a C-terminal HECT domain responsible for its E3 ligase activity [1]. In addition to WT, *HACE1* inactivation has been reported in multiple other tumor types, including non-Hodgkin’s lymphoma, as well as lung, ovarian, pancreatic, and prostate carcinomas [1, 3–14]. Consistent with this, genetic inactivation of *HACE1* in mice leads to the development of multiple late-onset tumors, including sarcomas, breast, lung, and other carcinomas, as well as lymphomas [3]. Recently, HACE1 was described as a potential tumor suppressor gene in osteosarcoma [13], where it was found to inhibit growth, as well as invasive and metastatic capacity in vivo [13]. However, the mechanism whereby HACE1 elicits these anti-tumorigenic effects remain incompletely characterized.

The most well-characterized E3 ligase target of HACE1 is RAC1 [10, 15–18]. We previously reported that HACE1 targets RAC1 for degradation when the latter is localized to the membrane nicotinamide adenine dinucleotide phosphate (NADPH) oxidase holoenzyme, inhibiting the generation of de novo reactive oxygen species (ROS) by RAC1-dependent NADPH oxidases [15]. RAC1 was originally identified as an oncogene promoting cancer cell survival and metastasis and essential for transformation by mutant KRAS [19]. Small GTPases (20–25 kDa) including the RAC1 cycle between their inactive inert GDP-bound and active GTP-bound states [20, 21]. Upon activation, interaction with effector proteins leads to stimulation of downstream signaling pathways that can impact cell migration and invasion [22–24].

Hypoxia-inducible factor-1alpha (HIF1α) is a transcription factor that is a key regulator of metastasis [25, 26]. HIF1α is induced following the onset of hypoxic stress, where it transcriptionally activates a wide array of genes that are important for the re-programming of multiple pathways that impact cell survival under hypoxia, such as angiogenesis [27–29]. Enhanced HIF1α levels have been reported in both low-grade and advanced tumors, suggesting a crucial role for HIF1α in oncogenesis [30–34]. Previous reports have shown that RAC1 is activated by stress stimuli including hypoxia [35, 36]. In addition, HIF1α induction under hypoxia requires activation of RAC1 [36–39]. Despite these established relationships between HACE1 and RAC1, and RAC1 and HIF1α, potential links between HACE1 and HIF1α have not been investigated.

In this study, we explore whether HACE1 influences HIF1α levels. We establish that HACE1 is induced at the mRNA and protein level under hypoxia. HACE1 reduces HIF1α accumulation in an E3 ligase dependent manner by reducing active RAC1 levels. In line with its putative tumor-suppressive role, the absence of HACE1 correlated with enhanced HIF1α protein levels in WT tissues compared to the patient-matched normal kidneys. Together, these data provide additional mechanistic insights into the tumor suppressor activity of HACE1, namely through the regulation of HIF1α.

## Results

### HACE1 expression is induced under diverse forms of cell stress

**Fig. 1:**
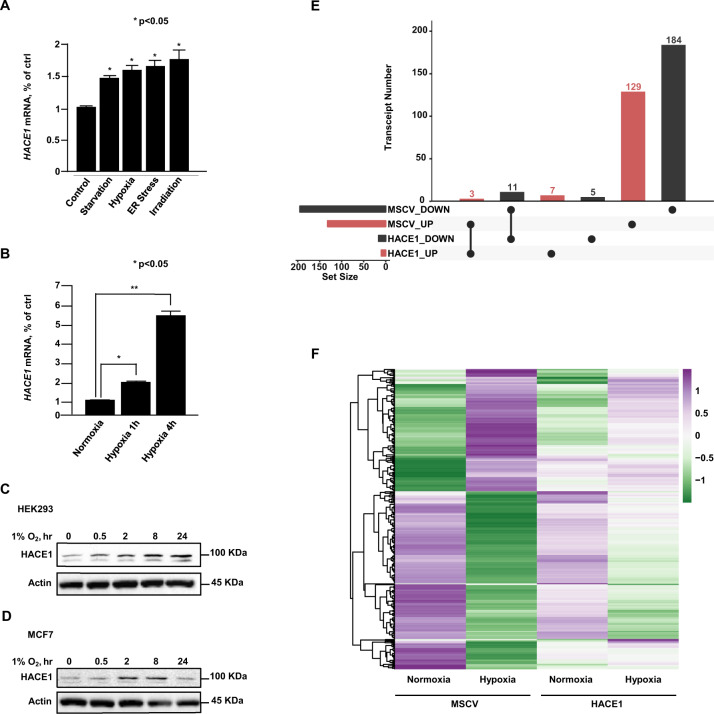
HACE1 expression is induced under diverse forms of cellular stress. **A** Relative expression levels of HACE1 mRNA under control and the indicated cellular stress conditions, as determined by qPCR. Data shown represent mean ± SD, *n* = 2 (*P < 0.05). **B** HEK293 cells were exposed to 1% O_2_ for the indicated times, followed by qPCR to determine HACE1 mRNA levels. Data shown represent mean ± SD, *n* = 3 (*P < 0.05; **P < 0.01). **C** Endogenous levels of HACE1 protein were examined in HEK293 cells cultured under 1% O_2_ for the indicated times by Western blotting. Actin was used as a loading control. **D** Protein levels of HACE1 increase under hypoxia. Endogenous levels of HACE1 protein were examined in MCF7 cells cultured under 1% O_2_ for the indicated times and lysates analyzed by Western blotting. Actin was used as a loading control. **E** UpSet plot shows a number of differentially expressed genes in HEK293 cells expressing HA-HACE1 or vector alone cells cultured under hypoxia versus normoxia. Differentially regulated genes were defined as having a fold difference of two or greater and an FDR < 0.05. Bars show the number of genes upregulated (red) and downregulated (black) during hypoxia, for HACE1 and vector alone cells. **F** The heatmap depicts the Z-score expression of 339 genes that show differential expression (fold difference of two or greater and an FDR < 0.05) in HA-HACE1 or vector alone cells during hypoxia.

It has previously been suggested that HACE1’s tumor suppressor activity may be linked to cellular stress responses [3]. To further investigate this possibility, we examined *HACE1* expression in HEK293 cells subjected to prototypical stress forms, namely short-term nutrient deprivation, hypoxia, endoplasmic reticulum (ER) stress, and γ-irradiation, as described in “Experimental procedures”. *HACE1* mRNA expression was increased by each of these stress forms, as shown in Fig. 1A. Since we previously studied HACE1 functions under oxidative stress [15], we explored its role during hypoxia for the present study. Under hypoxia, *HACE1* mRNA expression increased significantly and in a temporal manner (Fig. 1B). This was recapitulated at the protein level, whereby HACE1 increased in a time-dependent manner in hypoxia-exposed HEK293 and MCF7 breast cancer cells (Fig. 1C, D). We, therefore, further probed the consequences of *HACE1* induction in response to hypoxia. We performed Affymetrix gene expression profiling to compare vector alone control cells (MSCV) and HEK293 cells overexpressing HACE1 under normoxic versus hypoxic conditions (Fig. 1E, F, see Fig. S1 for relative HACE1 expression). In control cells exposed to normoxia and hypoxia, clear patterns of differential gene expression were observed (*n* = 327 genes with fold change > 2 and FDR < 0.05). This response was largely muted in HACE1 overexpressing cells, where only a subset of 26 of the above 327 genes met the same differential expression criteria (Fig. 1E and Table S1). Overlap of these differential expression sets revealed that only 14 genes (3 upregulated; 11 downregulated) were shared, suggesting that HACE1 overexpression mitigates the cellular response to hypoxia. This effect was clearly visible when comparing gene expression profiles under hypoxia vs. normoxia, where differentially expressed clusters in control cells under stress were reduced in cells overexpressing HACE1 (Fig. 1F). Together, these data indicate that *HACE1* is induced in response to diverse cellular stress and mitigates the transcriptional response under hypoxic conditions.

### HACE1 reduces HIF1α accumulation under hypoxia

**Fig. 2:**
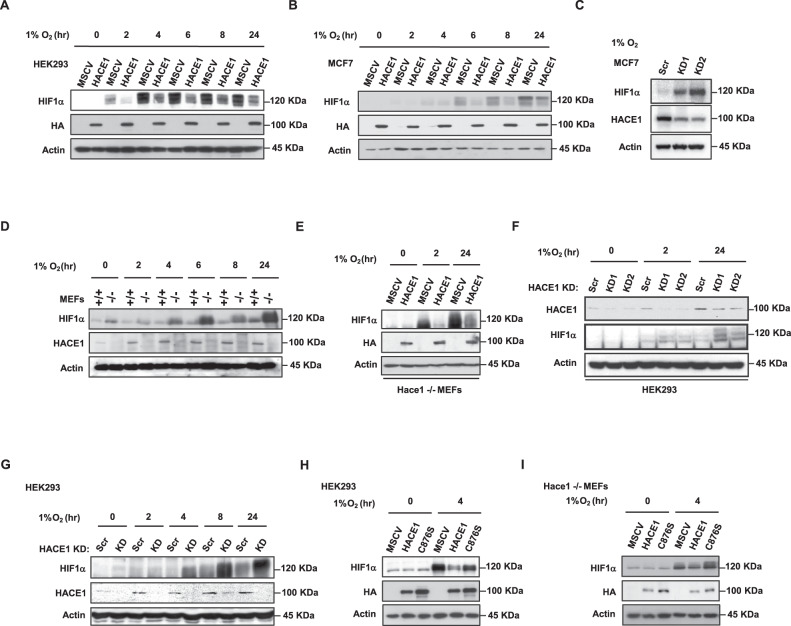
HACE1 blocks HIF1α accumulation. **A** HA-tagged HACE1 was overexpressed in HEK293 cells, and cells were exposed to 1% O_2_ for the indicated times. HIF1α and HA-HACE1 protein levels were examined by Western blotting. **B** HA-tagged HACE1 was overexpressed in breast cancer MCF7 cells, and cells were exposed to 1% O_2_ for the indicated times. HIF1α and HA-HACE1 protein levels were examined by Western blotting. **C** Transient knockdown of HACE1 using two individual siRNAs in MCF7 cells. Cells were exposed to 1% O_2_ for 4-h. HIF1α and HACE1 protein levels were examined by Western blotting. **D** *Hace1*^–/–^ and wild-type MEFs were exposed to 1% O_2_ for various times as indicated. Whole protein lysates were collected for immunoblotting with HIF1α and HACE1 antibodies. Actin was used as a loading control. **E** HA-tagged *HACE1* was expressed in *Hace1*^–/–^ MEFs using a retrovirus system. Cells were exposed to 1% O_2_ for 2- or 24-h. HIF1α protein levels were examined by Western blotting. **F** Transient knockdown of HACE1 using two individual siRNAs in HEK293 cells. Cells were exposed to 1% O_2_ for the times as indicated. HIF1α and HACE1 protein levels were examined by Western blotting. **G** Stable knockdown of HACE1 using shRNAs in HEK293 cells. Cells were exposed to 1% O_2_ for the times indicated. HIF1α and HACE1 protein levels were examined by Western blotting. **H** HEK293 overexpressing HA-tagged wild-type HACE1 or ligase dead HACE1-C876S were exposed to 1% O_2_ for 4-h and analyzed by Western blotting for HIF1α and HA-HACE1. **I** MEFs overexpressing HA-tagged WT HACE1 or ligase dead HACE1-C876S were exposed to 1% O_2_ for 4-h and analyzed by Western blotting for HIF1α and HA-HACE1.

Since HIF1α is a key transcription factor induced under hypoxia, transcriptionally activating a wide array of genes that are important for the cytoprotective re-programming of tumor cells under hypoxia [30, 40], including many in Table S1, we decided to examine potential relationships between HIF1α and HACE1. We first ectopically expressed *HACE1* in HEK293 and MCF7 cells and examined temporal HIF1α protein levels under hypoxia (1% O_2_). Overexpression of *HACE1* reduced HIF1α accumulation under hypoxia (Fig. 2A, B). Knockdown of HACE1 using siRNAs reversed this trend, whereby HIF1α levels increased under hypoxia (Fig. 2C). Next, wild type (*wt*) and *Hace1*^–/–^ mouse embryonic fibroblasts (MEFs) were exposed to hypoxia and cellular HIF1α levels were determined by Western blotting. Hace1 genetic inactivation led to a dramatic accumulation of HIF1α over 24 h compared to controls (Fig. 2D). Reintroduction of hemagglutinin (HA)-tagged *HACE1* into *Hace1*^–/–^ MEFs repressed HIF1α accumulation (Fig. 2E). In agreement with these observations, siRNAs targeting *HACE1* in HEK293 cells increased HIF1α accumulation compared to scrambled control siRNAs (Fig. 2F). In HEK293 cells with stable shRNA *HACE1* knockdown, HIF1α accumulation was also increased under hypoxia compared to controls (Fig. 2G). Together, these findings indicate that HACE1 regulates the level of the critical stress response and pro-oncogenic proteins, HIF1α under hypoxia. To determine if HACE1 requires its E3 ligase activity to reduce HIF1α levels, we ectopically expressed the previously described ligase dead HACE1-C876S mutant (Fig. S2) [1, 3] in HEK293 cells and *Hace1*^–/–^ MEFs. However, HACE1-C876S was unable to reduce HIF1α levels in HEK293 cells and *Hace1*^–/–^ MEFs (Fig. 2H, I). This indicates that the observed effects are dependent on the E3 ligase activity of HACE1.

### HACE1 reduces the half-life of HIF1α under hypoxia

**Fig. 3:**
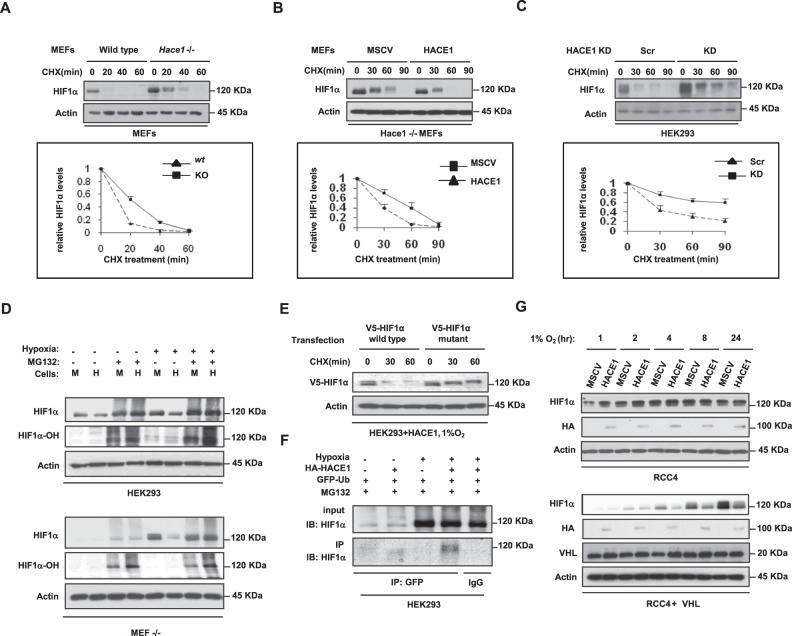
HACE1 leads to decreased stability and increased proteasome-dependent degradation of HIF1α. **A** *Hace1*^–/–^ and wild-type MEFs were cultured under 1% O_2_ for 4-h to accumulate HIF1α protein (0 min). Cells were then treated with cycloheximide (CHX) for the indicated time points to block protein synthesis, and lysates were analyzed for HIF1α levels by Western blotting. The graph shows quantified HIF1α protein amounts normalized to actin levels. **B** *HACE1* was expressed in *Hace1*^–/–^ MEFs by retroviral transduction. Cells were then treated under hypoxia with CHX as in Fig. 2A and similarly analyzed for HIF1α protein levels. **C** HEK293 cells stably transduced with HACE1 shRNAs (KD) or the scrambled control (Scr) were exposed to 1% O_2_ for 4-h and were treated with CHX for the indicated times. **D** HEK293 cells stably overexpressing HA-tagged HACE1 or vector alone were grown under 21% (normoxia) or 1% O_2_ for 2-h followed by treatment with the proteasome inhibitor MG132 for a further 2-h. Total, as well as the hydroxylated HIF1α levels, were examined by Western blotting using anti-HIF1α or anti-hydroxylated HIF1α (HIF1α-OH) antibodies, respectively. **E** Wild-type V5-tagged HIF1α, as well as mutant V5-HIF1α (P402A/P564A) that cannot be hydroxylated, was transiently transfected into HEK293 cells. Cells were culture under 1% O_2_ for 4-h to allow HIF1α protein to accumulate (0 min), followed by CHX treatment for the indicated times. Levels of exogenous HIF1α protein were analyzed by Western blotting using anti-V5 antibodies. **F** GFP-ubiquitin was transfected into HEK293 cells overexpressing HA-HACE1 or vector alone. Cells were cultured under 1% O_2_ for 3-h in the presence of MG132, followed by immunoprecipitation (IP) from whole-cell lysates using anti-GFP antibodies. Anti-HIF1α immunoblotting was performed to detect ubiquitin associated HIF1α proteins. IgG was used as an IP control antibody. **G** HA-HACE1 or vector alone constructs were stably overexpressed in RCC4 cells lacking VHL or RCC4 cells re-expressing wild-type VHL (RCC4 + VHL). Cells were exposed to 1% O_2_ for the indicated times and whole protein lysates were immunoblotted with anti-HIF1α or anti-HA antibodies for HIF1α and HACE1 levels.

HIF1α is an unstable protein that is strictly regulated by oxygen levels [41, 42]. To examine whether HACE1 affects the half-life of HIF1α, *Hace1*^–/–^ MEFs were exposed to hypoxia to allow HIF1α accumulation and subsequently treated with cycloheximide (CHX) to block new protein synthesis. The observed half-life of HIF1α in *wt* MEFs was short, with virtually all protein lost after just 20 min following CHX treatment (Fig. 3A). However, in *Hace1*^–/–^ MEFs, HIF1α protein signals were retained at both the 20- and 40-min time points, demonstrating an extended half-life (Fig. 3A). Re-expression of *HACE1* in knockout MEFs reversed this observation, restoring the HIF1α half-life to that found in *wt* cells (Fig. 3B). In agreement with these results, stable shRNA knockdown of *HACE1* in HEK293 cells also increased the stability of HIF1α compared to scrambled control cells (Fig. 3C).

To assess whether proteasome activity is required for HACE1-mediated reduction of HIF1α, *Hace1*^–/–^ MEFs stably expressing HA-Hace1 or vector alone (MSCV) were treated with the proteasome inhibitor, MG132, following pre-incubation in normoxic versus hypoxic conditions for 2 h. MG132 completely blocked the ability of HACE1 to reduce HIF1α levels under hypoxia (Fig. 3D). Proteasomal degradation requires hydroxylation on proline residues 402 and 564 of HIF1α by prolyl-hydroxylases (PHDs) [43]. We therefore examined the hydroxylation status of HIF1α and found that MG132 treatment led to the clear accumulation of the hydroxylated form of this protein under hypoxia in both MEFs and HEK293 cells (Fig. 3D). HACE1 over-expression resulted in enhanced levels of hydroxylated HIF1α in both cell types (Fig. 3D). Antibody specificity for hydroxylated HIF1α was confirmed using a PHD inhibitor, CoCl_2_ (Fig. S3), and these observations were further validated with a V5-tagged mutant HIF1α (P402A/P564A) that cannot be hydroxylated by PHDs nor degraded via the Elongin BC/Cul2/von Hippel Lindau factor (VHL) complex (ECV), an E3 ubiquitin ligase complex which targets the alpha subunit of HIF1α for ubiquitin-mediated degradation. [44–46]. V5-tagged *wt* or mutant HIF1α plasmids were transiently transfected into HEK293 cells stably expressing HA-*HACE1* and exposed to hypoxia (1% O_2_ for 4h) followed by a time-course of CHX treatment. *HACE1* overexpression reduced V5-HIF1α levels in *wt* cells but did not affect the mutant form (Fig. 3E), suggesting that hydroxylation is required for HACE1- mediated degradation.

To further investigate the link between HIF1α degradation and HACE1, we expressed GFP-tagged ubiquitin in HEK293 cells and examined the ubiquitylation status of HIF1α. Affinity purification using anti-GFP antibodies pulled down increased amounts of poly-ubiquitylated HIF1α in *HACE1* over-expressing cells compared to controls under 1% O_2_ (Fig. 3F). These findings indicate that HACE1 facilitates hydroxylation of HIF1α under hypoxia and triggers its ubiquitylation and degradation through the proteasome. Similar to HACE1-associated degradation of cyclin D1 [3], we found no evidence of direct ubiquitylation of HIF1α by Hace1 by in vitro assays, indicating that this process is likely indirect. Loss of function mutations of VHL protein, a key ECV component in renal clear cell carcinoma (RCC) results in elevated HIF1α levels [47, 48]. HA-HACE1 expression in RCC4 cells that express mutant VHL failed to reduce HIF1α levels when exposed to hypoxia (Fig. 3G). However, the effects of HACE1 on HIF1α were rescued in RCC4 cells re-expressing *wt* VHL (RCC4 + VHL), suggesting that HIF1α protein levels are regulated by HACE1 in a proteasome- and ECV-dependent manner. Together, these findings indicate that the impact of HACE1 on protein stability under hypoxia is due to increased protein degradation mediated by the hydroxylation and ubiquitylation status of HIF1α.

### HACE1 regulates HIF1α activity under hypoxia

**Fig. 4:**
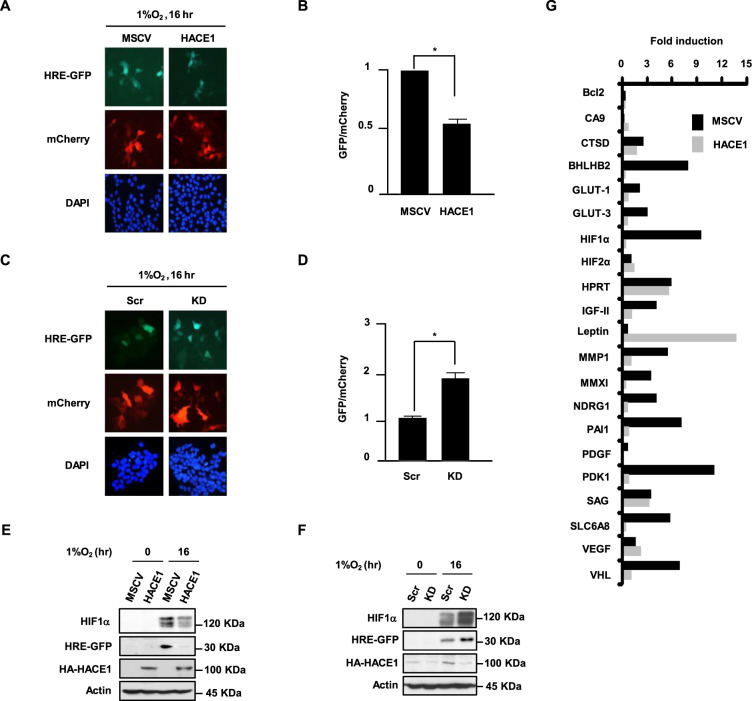
HACE1 regulates HIF1α activity under hypoxia. **A** HRE-GFP plasmids were transfected into HEK293 cells overexpressing HA-HACE1 or vector alone, along with a mCherry-encoding plasmid as a transfection control. Cells were then cultured under 1% O_2_ for 16-h. GFP and mCherry signals were examined by fluorescence microscopy. **B** Quantification of the relative levels of GFP positive cells in comparison to vector alone cells. (*P < 0.05; **P < 0.01). **C** An identical experiment as in (**A**) was performed using HRE-GFP expressing HEK293 after transfection with HACE1-targeting (KD) or the scrambled control shRNAs (Scr). **D** Quantification of the relative levels of GFP positive cells in comparison to vector alone cells (*P < 0.05; **P < 0.01). **E** HRE-GFP plasmids were transfected into HEK293 cells overexpressing HA-HACE1 or vector alone, along with a mCherry-encoding plasmid as a transfection control. Cells were cultured under 1% O_2_ for 16-h. HIF1α, HRE-GFP, and HA-HACE1 protein levels were examined by Western blotting. **F** HRE-GFP plasmids were transfected into HEK293 cells after transfection with HACE1-targeting (KD) or the scrambled shRNAs (Scr), along with a mCherry-encoding plasmid as a transfection control. Cells were then cultured under 1% O_2_ for 16-h. HIF1α, HRE-GFP, and HA-HACE1 protein levels were examined by Western blotting. **G** RCC4 + VHL cells expressing HA-HACE1 or vector alone were exposed to 1% O_2_ for 2-h. Total RNA was isolated, converted to cDNA in the presence of biotin-dUTP, and hybridized to gene-specific oligonucleotides in a 96-well plate. The captured cDNA was then detected with streptavidin-HRP on a microplate luminometer. Fold induction under hypoxia (compared to normoxia) is shown on the *y*-axis.

HIF1α binds to hypoxia response element (HRE) sequences in promoters of its target genes and regulates their expression [40, 49, 50]. To examine whether HACE1 reduces HIF1α functional activity under hypoxia, we transfected HEK293 control cells and cells stably expressing HA-HACE1 with an additional construct encoding GFP driven by an HRE-containing promoter (HRE-GFP) and cultured the resulting populations under hypoxia for 16-h. As a transfection control, a mCherry protein-encoding plasmid was co-transfected. Overexpression of HACE1 significantly reduced HRE-GFP signals under hypoxia (Fig. 4A, B). Similarly, HACE1 knockdown enhanced HRE-GFP expression compared to scrambled controls (Fig. 4C, D). The results of the HACE1 overexpression and knockdown were confirmed by Western blotting (Fig. 4E, F), indicating that HACE1-mediated reduction of HIF1α reduces its ability to regulate transcription of its target genes. Using cDNA hybridization, we probed the effects of HACE1-mediated control on the expression of known HIF1α target genes in RCC4 + VHL cells (i.e., HACE1 overexpression vs. vector alone), which were exposed to hypoxia for 3 h. HACE1 inhibited expression of many HIF1α target genes in the array; specifically, expression of genes involved in glucose metabolism (*PDK1, Glut-1*, and *Glut-3*) metastasis (*MMP1*, *MMX1*, and *PAI-1*), or signal transduction (*PDGF*) were all reduced by HACE1 (Fig. 4G). Notably, *VHL* mRNA expression was also reduced under hypoxia in HACE1 overexpressing cells, even though no changes in VHL protein levels were observed in our previous experiments (see Fig. 3G), potentially reflecting negative feedback regulation at the mRNA level. Together, these results demonstrate that HACE1-mediated regulation of HIF1α levels results in dynamic transcriptional changes in HIF1α targets regulated in response to hypoxia.

### Loss of HACE1 expression correlates with increased HIF1α expression in WT and sarcoma tissues

**Fig. 5:**
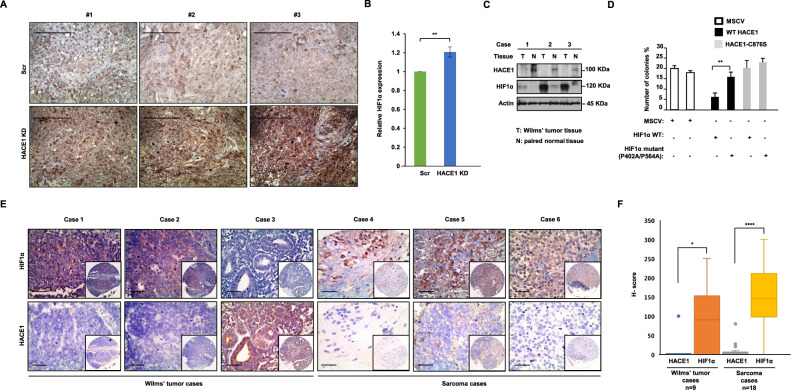
Loss of HACE1 expression correlates with increased HIF1α expression in Wilms’ tumor tissues. **A** Immunohistochemistry for HIF1α conducted on the paraffin blocks from nude mice injected with HEK293 cells with stable HACE1 knockdown or non-targeting shRNA used in this study, generated as described previously [3]. The scale bar is 100 μm. **B** Quantification of A was conducted respectively using ImageJ software and data represented as average value ± SEM for *n* = 15 HPFs in 3 tumors/group (*P < 0.05; **P < 0.01). **C** Whole-cell lysates from three cases of Wilms’ tumor (WT) with paired normal kidney (N) were subjected to Western blotting with antibodies endogenous HACE1 and HIF1α as indicated. **D** Constructs encoding wild type (*wt*) or HIF1α-P402A/P564A (non-OH-HIF1α) were expressed in SKNEP1 Ewing tumor cells stably expressing *wt* HACE1 or ligase dead HACE1-C876S as indicated. Cells were seeded in soft agar and grown for 14 days. Colonies were then counted as a percentage of seeded cells, using an inverted microscope. Data shown represent means ± SD, *n* = 3. (*P < 0.05; **P < 0.01). **E** Immunohistochemistry (IHC) of HIF1α and HACE1 were conducted on serial sections of TMAs consistent with Wilms’ tumors (9 cases) and sarcomas (18 cases) provided by Children Hospital of Philadelphia. Representative IHC images from matched patient samples on serial histological sections were shown. Scale bar = 100 μm. **F** Box plot showing H‐scores (staining intensity × percentage) for HIF1α and HACE1, along with relative sample sizes for each group based on the IHC analysis in (**E**) was shown. The cohort is grouped according to the tumor type. (*P < 0.05; **P < 0.01; ***P < 0.001; ****P < 0.0001).

To investigate whether HACE1 decreases HIF1α levels in vivo, we analyzed formalin-fixed paraffin-embedded (FFPE) tissues obtained from nude mice bearing xenografted HEK293 cells with or without stable *HACE1* knockdown, by immunohistochemistry (IHC) for HIF1α expression, as described previously [3]. In agreement with our in vitro observations, *HACE1* knockdown led to increased levels of HIF1α levels in tumor tissues (Fig. 5A, B). To probe the pathophysiological significance, we investigated the relative levels of HACE1 and HIF1α protein in WT cases compared with patient-matched normal kidney adjacent to tumor tissues. Consistent with our previous findings [3], HACE1 protein levels were reduced in all tumor cases tested (*n* = 3) compared to the patient-matched normal kidney, while HIF1α levels were correspondingly increased (Fig. 5C). To confirm that HACE1 tumor suppressor activity is linked to its ability to reduce HIF1α levels, we performed soft agar colony assays using the SKNEP1 human Ewing sarcoma cell line that lacks endogenous expression of HACE1 [1]. Ectopic expression of HACE1 in these cells was previously shown to significantly reduce in vitro soft agar colony formation [3]. As shown in Fig. 5D, HACE1 also significantly reduced colony formation in *wt* HIF1α expressing cells compared to parental SKNEP1 cells, while HACE1-C876S failed to do so. In cells expressing V5-tagged mutant HIF1α (P402A/P564A) in which HIF1α cannot be hydroxylated nor degraded by ECV, no changes were observed in colony formation in the presence of HACE1. To further assess the link between HACE1 and HIF1α in human tumors, tissue microarrays (TMAs) consisting of WT (9 cases) and different childhood sarcomas (18 cases, including 5 Ewing sarcoma cases, 3 alveolar rhabdomyosarcoma cases, 4 embryonal rhabdomyosarcoma cases, and 6 synovial sarcoma cases, which were analyzed collectively due to the limited case numbers) were subjected to IHC for HACE1 and HIF1α expression. A significant correlation was observed between high HIF1α and low HACE1 expression in both tumor cohorts (Fig. 5E, F), highlighting the inverse relationship between HACE1 and HIF1α levels in vivo. Together, these findings validate the role of HACE1 as a negative regulator of HIF1α activity in vivo.

### HACE1 blocks HIF1α accumulation under hypoxia through degradation of RAC1

**Fig. 6:**
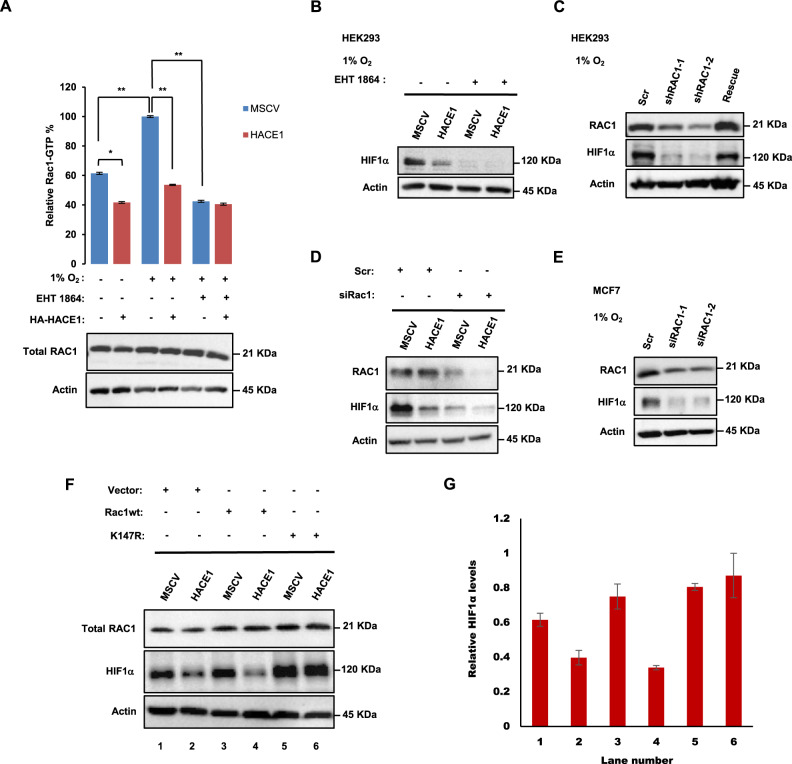
HACE1 blocks HIF1α accumulation under hypoxia in a RAC1 dependent manner. **A** GTP bound RAC1 levels are shown using RAC1 G-LISA Activation Assay Kit in the cells stably expressing HACE1 or the empty vector controls under normoxia or hypoxia in the presence or absence of EHT 1864. Total RAC1 levels are shown by Western blotting. (*P < 0.05; **P < 0.01). **B** HEK293 cells stably expressing HACE1 or the empty vector alone were treated (or not treated) with 20 μM EHT 1864 for 1-h, then subjected to hypoxia for 3-h. Protein levels of HIF1α were analyzed by Western blotting. **C** HEK293 cells with stable HACE1 KD were transfected with control shRNA (Scr) or one of two independent shRNAs targeting RAC1 (shRAC1). Rescue was generated by re-introducing pHAGE-RAC1 plasmid to the cells using a lentiviral transduction system. Cells were exposed to 1% O_2_ for 4-h and subjected to Western blotting. **D** HEK293 cells stably expressing HACE1 or the empty vector alone were transfected with control siRNA (Scr) or a siRNA targeting RAC1 (siRAC1), then subjected to hypoxia for 4-h before analysis of HIF1α expression by Western blotting. **E** MCF7 cells were transfected with control siRNA (Scr) or two individual siRNAs targeting RAC1 (siRAC1), then subjected to hypoxia for 4-h before being analyzed for HIF1α expression by Western blotting. **F** HEK293 cells stably expressing HACE1 or the empty vector alone were transfected with combinations of wild-type RAC1, RAC1-K147R, and vector alone, then subjected to hypoxia for 4-h before analysis of HIF1α and RAC1 expression by Western blotting. **G** Quantification of (**F**) was conducted respectively using ImageJ software and data represented as average value ± SEM.

Since RAC1 is required for activation of HIF1α [36–38], and RAC1 is the best-characterized ubiquitylation target of the HACE1 E3 ligase [10, 15–18], we wondered whether HACE1’s effects on HIF1α are mediated through RAC1. We found that RAC1 activation was higher under hypoxia than ambient conditions in HEK293 cells (Fig. 6A). While HACE1 overexpression reduced RAC1 activation under both ambient and hypoxic conditions, this reduction was proportionally greater under hypoxia, indicating that HACE1 can also inhibit RAC1 activation under hypoxia (Fig. 6A). To further test this connection, we incubated HEK293 cells ± ectopic *HACE1* overexpression with EHT1864, a selective inhibitor of RAC1 activation that traps RAC1 in an inactive state [51]. EHT1864 has been shown to inhibit RAC1 downstream signaling and cellular transformation by guanine nucleotide displacement, impairing RAC1 mediated functions in vivo [52]. EHT1864 treatment effectively prevented RAC1 activation in HEK293 cells, regardless of HACE1 overexpression, as expected (Fig. 6A). Moreover, EHT1864 blocked HIF1α accumulation under hypoxia in both control and HACE1 overexpressing cells (Fig. 6B), indicating that RAC1 activity is required for HIF1α induction under hypoxia in these cells.

**Fig. 7:**
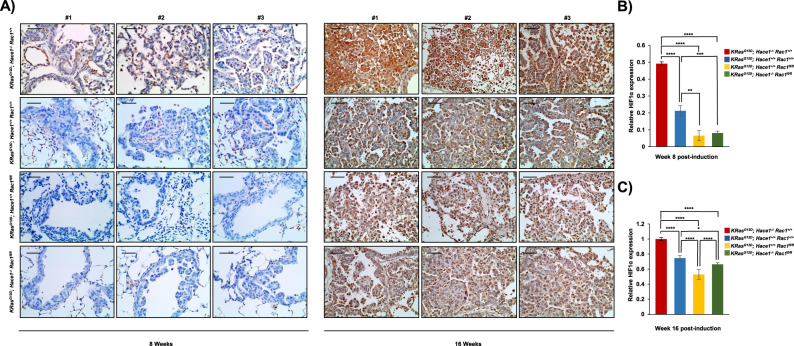
Genetic deletion of *Rac1* reversed the increased HIF1α expression of *Hace1*^*–/–*^ mice. **A** Immunohistochemistry for HIF1α conducted on the paraffin blocks from *the KRas*^*G12D*^-driven lung adenocarcinoma mouse model at weeks 8 and 16 after lung cancer induction for *KRas*^*G12D*^*Hace1*^*+/+*^*Rac1*^*+/+*^, *KRas*^*G12D*^*Hace1*^–/–^*Rac1*^*+/+*^, *KRas*^*G12D*^*Hace1*^*+/+*^*Rac1*^*fl/fl*^, and *KRas*^*G12D*^*Hace1*^–/–^*Rac1*^*fl/fl*^ mice, generated as described previously [53]. The scale bar is 100 μm. **B** Quantification of (**A**) (left panel) for 8 weeks after post-induction was conducted respectively using ImageJ software and data represented as average value ± SEM for *n* = 15 HPFs in three tumors/group. (*P < 0.05; **P < 0.01; ***P < 0.001; ****P < 0.0001). **C** Quantification of (**A**) (right panel) for 16 weeks after post-induction was conducted respectively using ImageJ software and data represented as average value ± SEM for *n* = 15 HPFs in three tumors/group. (*P < 0.05; **P < 0.01; ***P < 0.001; ****P < 0.0001).

Stable RAC1 knockdown using two independent shRNAs markedly reduced HIF1α levels under hypoxia, which could be rescued by ectopic RAC1 re-expression (Fig. 6C). Moreover, in HEK293 cells stably expressing ectopic HACE1, RAC1 knockdown further attenuated HIF1α accumulation (Fig. 6D). HIF1α accumulation was also reduced in MCF7 breast cancer cells under hypoxia when RAC1 was knocked down (Fig. 6E). Finally, to verify a link to HACE1 in these observations, we used a RAC1-K147R mutant that is resistant to HACE1 degradation, due to substitution of the RAC1 lysine residue targeted for ubiquitylation by HACE1 [10, 15]. In hypoxic HEK293 cells stably co-expressing ectopic HACE1 along with vector alone, RAC1-K147R, or wt RAC1, HACE1 was able to block HIF1α accumulation in control and wt RAC1 expressing cells (Fig. 6F, G; lanes 2 and 4). In contrast, cells expressing HACE1-resistant RAC1-K147R retained HIF1α accumulation under hypoxia even in the presence of HACE1 overexpression (Fig. 6F, G; lanes 5 and 6). To investigate this process in vivo, we used a described previously mouse lung cancer model of *Hace1* inactivation in which deletion of *Rac1* and expression of oncogenic KRas^G12D^ can be simultaneously induced by Adeno-Cre administration [3, 53]. IHC was performed to assess HIF1α expression in FFPE lung tumor tissues obtained from 8 and 16-week-old *KRas*^*G12D*^*Hace1*^*+/+*^*Rac1*^*+/+*^, *KRas*^*G12D*^*Hace1*^–/–^*Rac1*^*+/+*^, *KRas*^*G12D*^*Hace1*^*+/+*^*Rac1*^*fl/fl*^, and *KRas*^*G12D*^*Hace1*^–/–^*Rac1*^*fl/fl*^ mice. In agreement with our in vitro observations, deletion of *Hace1* alone (i.e. *KRas*^*G12D*^*Hace1*^–/–^*Rac1*^*+/+*^) resulted in elevated HIF1α levels compared to *KRas*^*G12D*^*Hace1*^*+/+*^*Rac1*^*+/+*^ controls, while genetic inactivation of *Rac1* alone (i.e. *KRas*^*G12D*^*Hace1*^*+/+*^*Rac1*^*fl/fl*^*)* decreased HIF1α levels compared to controls (Fig. 7A–C). *KRas*^*G12D*^*Hace1*^–/–^*Rac1*^*fl/fl*^ mice showed a significant reduction in HIF1α levels compared to *KRas*^*G12D*^*Hace1*^–/–^*Rac1*^*+/+*^ mice at both weeks 8 and 16 post-induction, and comparable HIF1α levels to tumors of *KRas*^*G12D*^*Hace1*^*+/+*^*Rac1*^*fl/fl*^ mice at both time points (Fig. 7A–C). Together, these data support a model in which HACE1 blocks HIF1α accumulation in a RAC1-dependent manner.

## Discussion

To better understand the regulatory mechanisms that underpin stress adaptation in cancer cells, we examined how the previously identified tumor suppressor HACE1 might function in such adaptive processes, given its previous links to cell stress [1, 3]. We observed consistent increases in *HACE1* mRNA expression under diverse stress conditions, highlighting HACE1 as a potentially broad stress response factor, in keeping with in vivo consequences of its genetic inactivation in mice, in which *Hace1* loss leads to the development of multiple late-onset tumors [3]. Here we show that hypoxia leads to marked increases in HACE1 at both the mRNA and protein levels, and surprisingly, that this is associated with loss of HIF1α protein. HIF1α induction in response to hypoxia is a well-established hallmark of cancer cell responses to stress, driving the transcription of factors essential for cell survival and ultimately dissemination under low oxygen conditions [27, 54, 55]. The ability to reduce HIF1α levels provides a plausible mechanism for the anti-tumorigenic activity of HACE1, including potential roles in blocking metastasis, as it was recently shown that HACE1 overexpression completely eliminated detectable lung metastases of osteosarcoma cells in mice [13].

We found that HACE1 did not appreciably alter *HIF1A* mRNA expression, but instead reduced HIF1α protein stability. Ligase dead HACE1-C876S was unable to reduce HIF1α levels in the models tested, indicating that this activity is dependent on HACE1 E3 ligase activity. However, we were unable to show that HACE1 directly targets HIF1α for ubiquitylation and degradation. Notably, HACE1 was unable to block the accumulation of a HIF1α mutant with substitution of proline residues 402 and 564, sites of HIF1α hydroxylation by PHDs [43], and critical for HIF1α degradation by the canonical ECV E3 ligase complex that targets HIF1α [44–46]. We found that HIF1α degradation still requires the ECV complex, as overexpression of HACE1 in RCC4 cells with mutant VHL was unable to block HIF1α accumulation unless the cells were engineered to re-express *wt* VHL. This suggests indirect targeting of HIF1α by HACE1. Interestingly, the kinetics of HIF1α accumulation following ectopic expression of HACE1 varied among the different cell lines tested. This might reflect differences in levels or isoforms of PHDs that hydroxylate HIF1α, or members of the ECV E3 ligase complex, that are expressed in each cell line. Moreover, levels of Fe^2+^, 2-oxyglutarate, ascorbate, and other metabolites can also influence HIF1α degradation, potentially also perturbing the kinetics of HIF1α degradation [56, 57].

A number of studies report that the upregulation of HIF1α in response to cellular stress is dependent on activation of the small Rho GTPase, RAC1 [36–38]. RAC1 has previously been identified as a proto-oncogene, where its activation leads to increased cell migration and invasion [22, 23]. In addition, RAC1 can be activated by stress stimuli such as hypoxia [35, 36]. HACE1 controls cell migration in different cell types through degradation of active RAC1 at NADPH oxidase complexes [15], including in breast cancer cells, where HACE1 inhibits cell migration and invasion by targeting active RAC1 [58]. Therefore, we searched for links between HACE1, RAC1, and HIF1α, and found that HACE1 blocks HIF1α accumulation under hypoxia in a RAC1 dependent manner. RAC1 is a primary target of the E3 ligase functionality of HACE1, suggesting a potential regulation point for mediation of HIF1α regulation by HACE1. This observation is consistent with previous findings indicating that HACE1 binds to RAC1 with higher affinity under hypoxia than normoxia, leading to its proteasomal dependent degradation, counteracting RAC1-mediated cell migration and invasion in breast cancer [58]. Moreover, we found that a RAC1-K147R mutant that is resistant to HACE1-mediated degradation rescued the accumulation of HIF1α in response to hypoxia in HACE1 overexpressing cells.

We also tested EHT1864, a relatively selective RAC1 activation inhibitor, and found that it abolished HIF1α accumulation under hypoxia. RAC1 and Cdc42 GTPases are key signaling intermediates with important roles in cancer initiation and progression, and inhibitors of these proteins, including EHT1864, have shown promising preclinical efficacy [59]. While none of the inhibitors targeting these GTPases have been approved for cancer therapy to date, RAC1/Cdc42 inhibition as a strategy to overcome therapy resistance has been validated previously [59–62]. Consistently, stable co-knockdown of RAC1 in HACE1 deficient cells inhibits HIF1α accumulation in these cells, suggesting targeting of RAC1 is a potential mechanistic intervention point for targeted therapy in diverse cancer types. In vivo, we found that loss of *Hace1* markedly enhances HIF1α expression in KRas^G12D^-driven lung tumors, while in contrast, *Rac1* loss alone decreases HIF1α expression in this model. Simultaneous deletion of *Hace1* and *Rac1* was similar to the loss of *Rac1* alone and showed significantly reduced HIF1α levels compared to the loss of *Hace1* alone, further suggesting that the ability of HACE1 to reduce HIF1α accumulation is dependent on RAC1.

HACE1 was originally identified as a tumor suppressor based on loss of expression in WT. In the present study, we found that HACE1 expression was also lost in WT specimens but retained in matched normal kidney tissues derived from WT nephrectomy specimens. Moreover, we detected HIF1α protein expression only in tumor tissues from these paired specimens, verifying the clinical relevance of our findings and consistent with previous observations [3]. These findings were corroborated by IHC staining of TMAs containing additional WTs as well as childhood sarcomas, also demonstrating a strong inverse relationship between HACE1 HIF1α protein levels. Further mechanistic details of how the HACE1-RAC1 axis regulates HIF1α stability, and whether other components are involved in this regulation, remains to be elucidated and warrants additional investigation. For example, previous studies of HIF1α suggest a crucial role for ROS generation might be involved in the stabilization of HIF1α under hypoxia [29, 36, 63]. Since RAC1 increases de novo ROS generation, and is targeted by HACE1 at NADPH oxidases [15], regulation of HIF1α by RAC1 might involve deregulated ROS generation. Our work illuminates a previously unrecognized link between HACE1, its primary target RAC1, and the oncogenic driver HIF1α. As HIF1α is well-established to facilitate oncogenesis in a variety of cancers, the HACE1-RAC1 axis provides an attractive potential target for therapeutic intervention.

## Experimental procedures

### Reagents

Cycloheximide, MG132, and cobalt chloride (CoCl_2_) were obtained from Calbiochem (San Diego, CA). Antibodies and plasmids used in this study are described in Supplemental Experimental Procedures.

### Cell culture

RCC4 and RCC4-VHL cells were generous gifts from Dr. Michael Ohh (University of Toronto). Culture conditions for each cell line are described in Supplemental Experimental Procedures. The cell lines stably expressing MSCV, HA-HACE1, or the mutant HA-HACE1-C876S were generated as described [1]. Stable knockdown HACE1 cell lines were generated using the Block-iT Lentiviral RNAi kit (Invitrogen, Carlsbad, CA, USA) according to the manufacturer’s protocols as described [3]. Nutrient deprivation was performed using HBSS/HEPES solution for the indicated time points. Hypoxia treatment consisted of culturing in a hypoxia chamber (COY Laboratory Products, INC. Grass Lake, Michigan) at 1% O_2_, 5% CO_2_, and balanced with N_2_ at 37 °C. Cells were seeded in equal numbers and incubated under the same conditions, then randomized and categorized into control and experimental groups in all experiments conducted unless stated otherwise.

### Transfections

Transient plasmid transfections were performed using FuGene six reagent (Roche, Penzberg, DE) in a six-well plate, according to the user manual. Transfections of siRNAs were performed with 25 nM siRNA using RNAiMax (Invitrogen, Waltham, MA, USA). The following siRNAs were used in the study: control siRNA (C): (5′–3′) AUAUCGGCUAGGUCUAACA; Hace1-1 (H1): Hs_Hace1_1 (FlexiTube, Qiagen, Hilden, DE); Hace1–2 (H2): Hs_Hace1_4 (FlexiTube, Qiagen, Hilden, DE); Human Rac1 (R): Hs_Rac1_6 (FlexiTube, Qiagen, Hilden, DE); and FlexiTube GeneSolution GS5879 (FlexiTube, Qiagen, Hilden, DE).

### Immunoprecipitation and immunoblotting

Immunoprecipitation and immunoblotting were conducted using standard methods. Detailed procedures are described in the Supplemental Experimental Procedures.

### Immunohistochemistry

IHC on tissue sections obtained from the paraffin blocks generated previously by Kogler et al. [53] and Zhang et al. [3] was conducted on a Ventana Discovery XT system. All IHC analysis was conducted blindly by two independent pathologists. Detailed procedures are described in the Supplemental Experimental Procedures.

### RAC1 G-LISA activation assay

The G-LISA RAC1 activation kit (Cat# BK126 Cytoskeleton, Denver, CO, USA) was performed according to the user manual to determine active (GTP bound) RAC1 levels. Detailed procedures are described in the Supplemental Experimental Procedures.

### Clinical patients

The three cases of WT with matching normal kidney were collected from the Children’s Oncology Group (COG) WT tissue bank, and generously supplied by Dr. Paul Grundy, Cross Cancer Center, Edmonton, Alberta.

### Statistical analysis

All statistical analysis was conducted using a Student’s two-tailed *T*-test, unless otherwise indicated, with *p*-values < 0.05 being considered as being statistically significant.

## Supplementary information

Supplemental Data

Supplemental Table S1
