# Supplementary material for: HACE1 blocks HIF1α accumulation under hypoxia in a RAC1 dependent manner
Source: Oncogene. 2021 Feb 18;40(11):1988–2001. doi: 10.1038/s41388-021-01680-1 (PMC7979542; doi:10.1038/s41388-021-01680-1)
Supplement: Supplementary file 1 — Supplemental Data [file 41388_2021_1680_MOESM1_ESM.pdf]

SUPPLEMENTAL DATA

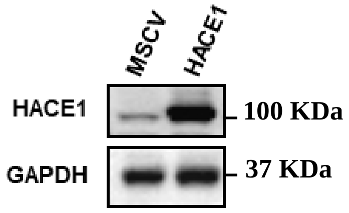

Figure S1. Endogenous levels of HACE1 protein in HEK293 cells stably expressing HACE1 and the empty vector alone

Whole protein lysates were collected to examine the levels of HACE1 protein in HEK293 cells stably expressing HACE1 and empty vector alone (MSCV). Protein levels of HACE1 were analyzed by Western blotting.

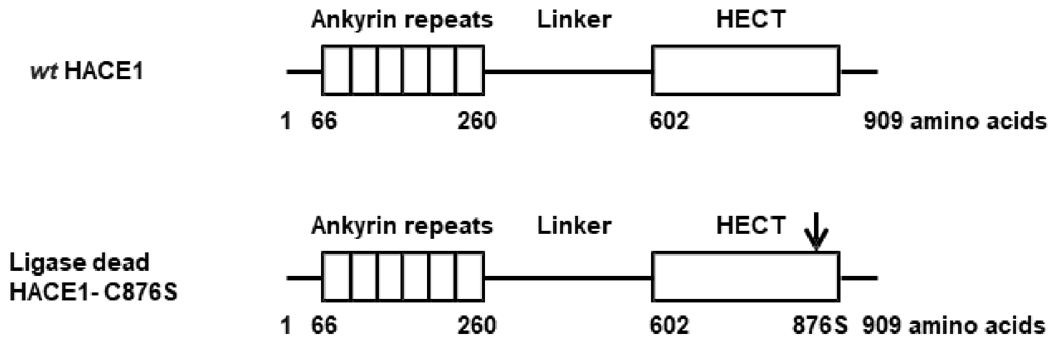

Figure S2. A diagram showing the structures of wild type and mutant HACE1 proteins

The structures of *wt* HACE1 and the ligase dead mutant HACE1 C876S proteins are shown.

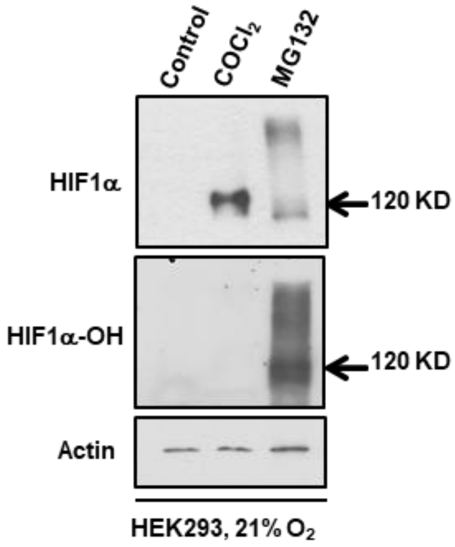

Figure S3. Specificity test for the anti-HIF1α-OH antibody

HEK293 cells were cultured under normoxia conditions in the presence of Cobalt Chloride ($CoCl_2$) or MG132 for 4-hours. The protein levels of HIF1α and hydroxylated HIF1α were examined by Western blotting.

Table S1. Genes differently expressed in HEK293 cells stably expressing HACE1 or MSCV under hypoxia vs normoxia

HEK293 cells expressing HA-HACE1 or vector alone were exposed to hypoxia (1% O_2) for 3-hours. For each cell line and culture condition, two independently isolated RNA samples were hybridized to Affymetrix GeneChip Human Exon 1.0 ST (HuEx 1.0) arrays. The criteria defined for selection of differentially expressed genes between hypoxia and normoxia in HEK293 HA-HACE1 and MSCV cells was based on fold-difference of at least 2 or greater and FDR <0.05. All data management and analysis were conducted using R software.

SUPPLEMENTAL EXPERIMENTAL PROCEDURES

Reagents

Antibodies used in this study include: anti-HA (Cat# MMS-101P-1000 (1.0 ml), Covance (Cedarlane), Ontario, CA); anti-GFP (Cat# MB600-308, Novus Biologicals, Centennial, USA); anti-ACTIN (Cat# 8457 (D7A8), Cell Signaling, Danvers, MA, USA); anti-HIF1α (Cat#10006421,Cayman Chemical, Ann Arbor, MI, USA); anti-Hydroxy-HIF1α (Pro564) (D43B5) (Cat#3434; Cell Signaling, Danvers, MA, USA);anti-HIF2α (Cat# 7096, Cell Signaling, Danvers, MA, USA); anti-HIF1β (Cat# ab2771, Abcam, Cambridge, UK); anti-RAC1 (Cat# 610650, BD Biosciences, San Jose, CA, USA), anti-HACE1 (Cat# ab133637; Abcam, Cambridge, UK); anti-GAPDH (Cat# 2118; Cell Signaling, Danvers, MA, USA); anti-VHL (Cat# 2738, Cell Signaling, Danvers, MA, USA). Monoclonal antibodies were selected using standard procedures and employed in this study. Anti-mouse IgG-HRP, anti-rabbit IgG-HRP, or anti-goat IgG HRP antibodies were from Santa Cruz Biotechnology (Dallas, TX, USA). EHT1864 is purchased from TOCRIS (Cat# 3872).

Plasmids used include: V5-HIF1α and mutant V5-HIF1α (P402A/P564A) are kind gifts from Dr. Thilo Hagen (National University of Singapore); HRE-GFP, is a gift from Dr. Peggy Olive from British Columbia Cancer Research Centre, Vancouver, BC. MSCV-HA, MSCV-HA-Hace1 and MSCV-HA-Hace1-C876S were cloned as described in Zhang et al. GFP-Hace1 and GFP Hace1-C876S were generated by cloning Hace1 and C876S into pEGFP-C1 (Clontech, Mountain View, CA, USA). GFP-Rac1wt, GFP-Rac-1v12 were generated as described in Castillo-Lluva et al. and GFP-Rac1-K147R was made by PCR-based nucleotide mutagenesis in GFP-Rac1. pHAGE-RAC1 plasmid was purchased from Addgene (Cat# 116783, Watertown, MA, USA).

Cell culture

HEK293 cells (ATCC) were maintained in Dulbecco's modified Eagle's medium (DMEM) containing 9% fetal bovine serum (FBS). Immortalized wild-type and HACE1-/- MEFs were prepared as described previously (Zhang et al., 2007) and cultured in DMEM containing calf serum. MCF7 cells (ATCC) were grown in RPMI medium supplemented with 9% FBS. RCC4 and RCC4-VHL cells are cultured in DMEM containing 10% FBS, 2 mM Glutamine and G418 0.5mg/ml.

Immunoprecipitation and immunoblotting

For immunoprecipitation, cells were lysed in Nonidet P-40 lysis buffer containing 100 mM NaCl, 5 mM MgCl2, 2 mM EDTA, 1 mM DTT, 10 mM Tris-HCl (pH 7.6), 0.5 % Nonidet P-40, 0,05% sodium deoxycholate, and 1 mM phenylmethylsulfonyl fluoride (PMSF). Cell debris was removed by centrifugation at 13,000 rpm for 20 min, and cell extracts (800 µg protein) were incubated with indicated antibodies overnight. Protein G- or A-Sepharose beads (Thermofisher, Waltham, MA, USA) (50 µl) were used to pull down antibody-protein complexes and were washed with lysis buffer for 3 times to remove non-specific binders. Bound proteins were eluted by boiling in Laemmli buffer for 5-minutes. Immunoblotting was performed using standard methods. Lysates (10–30 µg per lane) were separated with SDS-PAGE gels and transferred to nitrocellulose membranes. The proteins of interest were examined by blotting with respective antibodies overnight at +4C and anti-mouse or rabbit IgG-HRP for 1 hr at RT. All antibodies were used at a dilution of 1:1000 unless otherwise stated. Signals were detected using Enhanced Chemiluminescence (ECL).

Immunofluorescence microscopy

HEK293 cells expressing HA-tagged HACE1 or the vector alone were grown on coverslips. Cells were exposed to various stresses as described above and fixed with 3.5% paraformaldehyde. Cells were permeabilized with 0.5% Nonidet P-40 for 5-minutes and nonspecific signaling was blocked with 3% skim-milk for 10-minutes. HEK293 cells were transfected with plasmids encoding HRE-GFP and Cherry and then cultured under 1% O2 for 16 hrs. GFP and Cherry signals were examined by fluorescence microscopy using a Zeiss LSM 780 confocal microscope (Carl Zeiss, Thornwood, NY) and quantified using ImageJ software.

RAC1 G-LISA activation assay

25 μg total of protein was added wells pre-coated with RAC-GTP-binding protein. Then, the plate was incubated at 4°C for 30-minutes followed by incubation with 50 μl of anti-RAC1 (1/50 in Antibody Dilution Buffer) for 45-minute at RT. After rinsing 3 times with the wash buffer, the plate was incubated with a secondary antibody conjugated with HRP (1/100 in Antibody Dilution Buffer) for 45 min. Finally, 50 μl of HRP detection reagent was added to wells and incubated for 20-minutes. Lastly, 50 μl HRP stop solution was added to each well to stop the reaction and the absorbance was recorded at 490 nm.

HIF1α target gene hybridization assays

Hybridization assays were carried out using the Human HIF-regulated cDNA plate array system (Cat# AP-0111, Signosis BioSignal Capture, Santa Clara, CA, USA), according to according to the manufacturer's protocols. Briefly, RNA samples prepared from 1% O2 or normoxia treated HEK293 cells were reverse transcribed into cDNA in the presence of biotin-dUTP. The cDNA was then incubated with gene-specific oligonucleotides pre-coated in the individual wells of a 96-

well plate. The captured cDNA was detected with streptavidin-HRP with a dilution of (1:500) on a microplate luminometer. The data were analyzed following normalization to actin levels.

RNA isolation and qRT-PCR

Quantitative RT-PCR (qRT-PCR) was performed to assess HACE1 mRNA levels. Total RNA from cells was isolated with the RNeasy Mini Kit (Qiagen, Hilden, DE). Complementary DNA (cDNA) was synthesized from total RNA with the High-Capacity cDNA Reverse Transcription Kit (Applied Biosystems, Waltham, MA, USA). Quantitative PCR (qPCR) was performed using Fast SYBR™ Green Master Mix (Applied Biosystems, Waltham, MA, USA) on QuantStudio 6 Real-Time PCR Systems (Thermo Fisher, Waltham, MA, USA). GAPDH was used as the reference gene. The primers used for human HACE1 (forward: GGGCAGACAGCACTGCATGT, reverse: TTGCTCCTGATACATTTGGC) and GAPDH (forward: GTCTCCTCTGACTTCAACAGCG, reverse: ACCACCCTGTTGCTGTAGCCAA)

Soft agar colony assays

Soft agar assays were carried out as described (Zhang et al., 2007). SKNEP1 cells expressing wild-type or mutant HIF1α-P402A/P564A were suspended in soft agar and seeded in 6-well culture dish at a density of 45,000 cells per well. The cells were grown in a hypoxia chamber for 2 weeks. After colonies are stained, wells were imaged, and colony numbers were counted using ImageJ.

Immunohistochemistry

Antibodies used are HIF1α (Cat# NB100-134; Novus Biologicals, Centennial, USA) at a dilution of 1:150, HACE1 (Cat# 133637; Abcam, Cambridge, UK) at 1:300 with signal stain booster.

Quantitative analysis of IHC samples was conducted using ImageJ software. Data represented as average value ± SEM for n = 15 HPFs in 3 tumors/group.

Tissue microarrays (TMAs) from Children's Hospital of Philadelphia were prepared according to standard protocols and consisted of formalin-fixed, paraffin-embedded human tissue cores from primary tumors of Wilms' tumors (9 cases) and 18 cases sarcomas, including Ewing sarcoma (5 cases), alveolar rhabdomyosarcoma (3 cases), embryonal rhabdomyosarcoma (4 cases), and synovial sarcoma (6 cases). These were stained for HACE1 and HIF1α expression as above. Cores were scored for the percentages of cells positively staining for HACE1 and HIF1α as well as for staining intensities. For the latter, a 4-point scale (0–3+) was used according to standard methods.

Gene expression profiling

HEK293 cells expressing HA-HACE1 or vector alone were exposed to hypoxia (1% O2) for 3-hours. RNA samples were prepared using TRIZOL (Invitrogen, Waltham, MA, USA), and 5 µg of each RNA sample was used for microarray target synthesis and hybridization as described in the Affimetrix GeneChip manual (Affymetrix, Santa Clara, CA). For each cell line and culture condition, two independently isolated RNA samples (from different cell culture plates, i.e., biological replicates) were hybridized to Affymetrix GeneChip Human Exon 1.0 ST (HuEx 1.0) arrays. Raw data were read and processed by frozen RMA normalization (1). Only "core" probesets were retained for the downstream analysis. Differential expression between hypoxia and normoxia condition was computed by probe-level expression change averaging (PECA) procedure (2). The criteria defined for selection of differentially expressed genes between hypoxia and normoxia in HEK293 HA-HACE1 and MSCV cells was based on fold-difference of at least 2 or greater and FDR <0.05. All data management and analysis were conducted using R software.

Microarray data sets are available at http://www.ncbi.nlm.nih.gov/geo under accession code GSE25452.

SUPPLEMENTAL REFERENCES

1. McCall MN, Bolstad BM, Irizarry RA. Frozen robust multiarray analysis (fRMA). Biostatistics. 2010;11(2):242-53.
2. Elo LL, Lahti L, Skottman H, Kyläniemi M, Lahesmaa R, Aittokallio T. Integrating probe-level expression changes across generations of Affymetrix arrays. Nucleic acids research. 2005;33(22):e193.
